# Metformin alleviates adriamycin resistance of osteosarcoma by declining YY1 to inhibit MDR1 transcriptional activity

**DOI:** 10.1186/s40360-023-00685-8

**Published:** 2023-10-12

**Authors:** Bowen Wu, Peng Li, Eryue Qiu, Jian Chen

**Affiliations:** 1 Department of Orthopedics, Zhuzhou central hospital, 116 Changjiangnan Road, Tianyuan District, Zhuzhou, 412007 Hunan China; 2 Trauma center, Zhuzhou central hospital, Zhuzhou, 412007 Hunan China

**Keywords:** Osteosarcoma, Metformin, Adriamycin resistant, Yin Yang 1, Multi-drug resistance-1

## Abstract

Chemotherapy resistance hinders the successful treatment of osteosarcoma (OS) to some extent. Previous studies have confirmed that metformin (Met) enhances apoptosis induced by chemotherapeutic drugs, but the underlying mechanism remains unclear. To establish adriamycin (ADM)-resistant MG-63 (MG-63/ADM) cells, the dosage of ADM was progressively increased. The results of qRT-PCR and Western blotting demonstrated that the expression level of Yin Yang 1 (YY1) and multi-drug resistance-1 (MDR1) in MG-63/ADM cells were remarkably increased compared with those in MG-63 cells. Met dramatically enhanced ADM cytotoxicity and accelerated apoptosis of MG-63/ADM cells. Moreover, Met suppressed the expressions of YY1 and MDR1 in MG-63/ADM cells. YY1 promoted its transcriptional expression by directly binding to the MDR1 promoter. Furthermore, the effects of Met on ADM sensitivity in MG-63/ADM cells was reversed due to overexpression of YY1 or MDR1. Collectively, these findings suggested that Met inhibited YY1/MDR1 pathway to reverse ADM resistance in OS, providing a new insight into the mechanism of Met in ADM resistance of OS.

**Supplementary Information:**

The online version contains supplementary material available at 10.1186/s40360-023-00685-8.

## Introduction

Osteosarcoma (OS) has been reported as the most common primary bone malignancy in children and adolescents [1, 2]. OS usually occurs in distal femur, metaphyseal region of proximal humerus and proximal tibia [3]. OS is characterized by high invasiveness, high incidence of pulmonary metastasis and poor prognosis [4]. Chemotherapy is usually the preferred treatment for OS, despite its high cost, the high incidence of drug resistance and high side effects [5]. However, multidrug resistance is an important factor leading to poor prognosis and chemotherapy failure in patients with OS [6]. Some natural molecules play an important part in cancer chemoprevention, such as polyphenols, which exert their effects by regulating antioxidant, anti-inflammatory and apoptotic signal transduction pathways in the tumor microenvironment [7].

Metformin (Met) is a common hypoglycemic drug which suppresses the growth of various malignant tumors. As shown by Sanjay Goel, et al., Met has anti-inflammatory effects, anti-cancer activity, antimicrobial activity and immunosuppressive properties, regulates oxidative balance, helps in weight loss, and exerts neuroprotective effects [8]. Met improves the 5-year survival rate, prognosis and postoperative quality of tumor patients, and reduces the recurrence rate of tumor [9, 10]. Previous studies have shown that Met increased the sensitivity of OS cells to adriamycin (ADM) and cisplatin [11], but the underlying mechanism remains unclear. The P-glycoprotein (P-gp) protein encoded by multi-drug resistance-1 (MDR1) gene is the first confirmed transporter protein [12]. Up-regulation of P-gp lead to the efflux of chemotherapy drugs out of the cells, resulting in drug resistance, while Met reverses drug resistance in breast cancer cells by inhibiting the expression level of P-gp [13]. It has been reported that the expression level of P-gp is dramatically enhanced in chemoresistance tumor cells [14], and silencing P-gp may be an effective treatment to OS resistance. However, there are few reports that P-gp is involved in Met-mediated chemoresistance. Therefore, identifying the mechanism of Met sensitivity to ADM in OS cells may improve the prognosis of patients with OS.

Transcription factor Yin Yang 1 (YY1) regulates its transcriptional activity by directly targeting the promoters of downstream genes [15]. YY1 expression is up-regulated in OS tissues and cells, and the high expression of YY1 is closely associated with the poor prognosis of patients with OS [16]. It has been reported that YY1 directly binds to the multi-drug resistance-1 (MDR1) promoter to promote its expression [17]. Met treatment inhibits the expression of YY1 in myocardial cells [18]. However, there are few studied on the OS resistance of YY1 at present.

In this study, we presented the underlying mechanism of Met in ADM resistance of OS. We found that Met could enhance the killing effect of ADM on OS and inhibit cell proliferation. Moreover, Met regulated the transcription activity of MDR1 by down-regulating YY1, thereby reducing ADM resistance in OS. This study is the first to find that YY1 is a key molecule for Met to alleviate ADM resistance in OS, providing a new idea and direction for resolving chemoresistance in OS.

## Materials and methods

### Cell culture

Human OS cell line MG-63 was provided by American Type Culture Collection (USA). MG-63/ADM cells were established by stepwise increasing ADM dosage. MG-63 cells in logarithmic phase were inoculated into DMEM culture medium containing ADM. The drug concentration of ADM was increased gradually with a gradient of 0.1, 0.2, 0.5, 1.0, 2.0, 4.0 µg/mL. The MG-63/ADM resistant cells were obtained by stably growth at the concentration of 2.0 µg/mL ADM, and the whole induction cycle lasted for 70 days [19]. The cells were exposed to different concentrations of ADM (0, 2, 4, 8, 16, 32 and 64 µg/mL). MG-63 and MG-63/ADM cells were cultivated in DMEM medium supplemented with 10% FBS, 100 µg/mL streptomycin and 100 µg/mL penicillin (Thermo Fisher Scientific, USA) at 37 ℃ in a 5% CO_2_ atmosphere. According to previous study [20], low (1 mM), middle (3 mM) and high (10 mM) concentration of Met were added and incubated for 24 h at 37 ℃.

### Cell transfection

For transfection, cells (1.2 × 10^6^ cells/well) were seeded in 6-well plate. The YY1 and MDR1 overexpression vectors were generated through inserting full-length sequences into pcDNA3.1. The empty vector was used as control. Cell transfection was carried out using Lipofectamine 2000 reagent (Invitrogen, USA) as per the protocol.

### MTT assay

After treatment with different concentrations of ADM, MTT assay was conducted using an MTT kit (Sigma, USA) to assess cell viability. After incubation with 10 µL MTT reagent (0.5 mg/mL) for 4 h, 100 µL of DMSO (Sigma, USA) was added subsequently and incubated for 1 h. Microplate reader (BIO-RAD, USA) was used to measure the absorbance at 490 nm. The growth curve was plotted by calculating the half maximum inhibitory concentration (IC50) of each group.

### Apoptosis detection using flow cytometry

Cell apoptosis was evaluated by Flow cytometry using Annexin V-FITC/PI staining kit (Invitrogen, USA). The cells were trypsinized, washed with cold PBS buffer, and stained using 5 µL Annexin V-FITC and 10 µL PI solution. Subsequently, cell apoptosis rate was quantitatively assessed by FACScan flow cytometer (Beckman Coulter, USA). PI^−^ Annexin V^+^ and PI^+^ Annexin V^+^ were calculated for apoptosis rate.

### Luciferase activity assay

The wild type and mutant type sequences of MDR1 promoter containing YY1 binding sites were cloned into pGL3 vector (Promega, USA). Subsequently, cells (10^5^ cells/well) were incubated in 24-well plates and transiently co-transfected with YY1 overexpression vector and pGL3 (Promega) using Lipofectamine 3000 reagent (Invitrogen, USA). Luciferase activities in cell lysates were monitored using a luminometer (TD-20; Turner Designs, USA).

### Chromatin immunoprecipitation (ChIP) assay

ChIP assay was conducted in line with the instructions of MAGnify™ Chromatin Immunoprecipitation System (Invitrogen, USA). MG-63 cells were fixed with 1% formaldehyde, and sonicated to obtain DNA fragments. Appropriate anti-YY1 antibody (Abcam, USA) was added and incubated at 4℃ overnight. The anti-H3 and normal IgG were served as positive and negative control, respectively. After adding Magnetic beads (Millipore, USA) and vortexing, the complexes were digested with proteinase K and DNA was purified with magnetic beads. The sites of interest were amplified by PCR.

### Western blot assay

Total proteins were extracted for each cell using RIPA reagent (Beyotime, China), then the protein concentrations were measured using the BCA protein assay kit (Thermo Fisher Scientific, MA, USA). Equal amounts of proteins were loaded onto 10% SDS-PAGE for separation, and then electrically transferred to the PVDF membranes (Invitrogen, USA). The PVDF membranes were clipped according to the molecular weight of the target protein. The samples were blocked in 5% non-fat milk for 60 min. Subsequently, primary antibodies against YY1 (1:2000), P-gp (1:1000), Bax (1:1000) and Bcl-2 (1:1000), all purchased from Abcam (USA), were incubated overnight at 4℃. Then anti-rabbit IgG was applied as the secondary antibody. The immunoreactive bands were visualized by the electrochemical luminescence detection system as per the instructions. Each protein level was normalized with GAPDH as an internal control.

### Quantitative real-time polymerase chain reaction (qRT-PCR)

Total RNA was extracted according to TRIzol reagent (Invitrogen, USA) and RNA levels were quantified. Reverse transcription was conducted according to instructions of reverse transcription kit (Takara, China). The relative mRNA expression levels were detected by qRT-PCR assay using the SYBR Green Master PCR mix (Applied Biosystems, USA) as per the manufacturer’s protocol. GAPDH was utilized as the internal reference. The relative expression of mRNA was calculated by 2 ^–ΔΔCT^ method. The primers were as follows:

YY1 (F): 5’-ATACCTGGCATTGACCT-3’;

YY1 (R): 5’-TGAGGGCAAGCTATTGT-3’;

MDR1 (F): 5’-ATGACCAGGTATGCCTATTATTAC-3’;

MDR1 (R): 5’-CACATCAAACCAGCCTATCTC-3’;

GAPDH (F): 5’-CCAGGTGGTCTCCTCTGA-3’;

GAPDH (R): 5’-GCTGTAGCCAAATCGTTGT-3’.

### Statistical analysis

GraphPad Prism (GraphPad, San Diego, CA, USA) was used for relevant data analysis. All data were based on 3 times replicates. The values were presented as mean ± standard deviation (SD). Student’s *t-test* was adopted for pairwise comparison, and one-way ANOVA with *Turkey’s post hoc test* was conducted for multi-group comparison. *P* < 0.05 represented statistically significant difference.

## Results

### Relative expressions of YY1 and MDR1 in ADM resistant OS cells

**Fig. 1:**
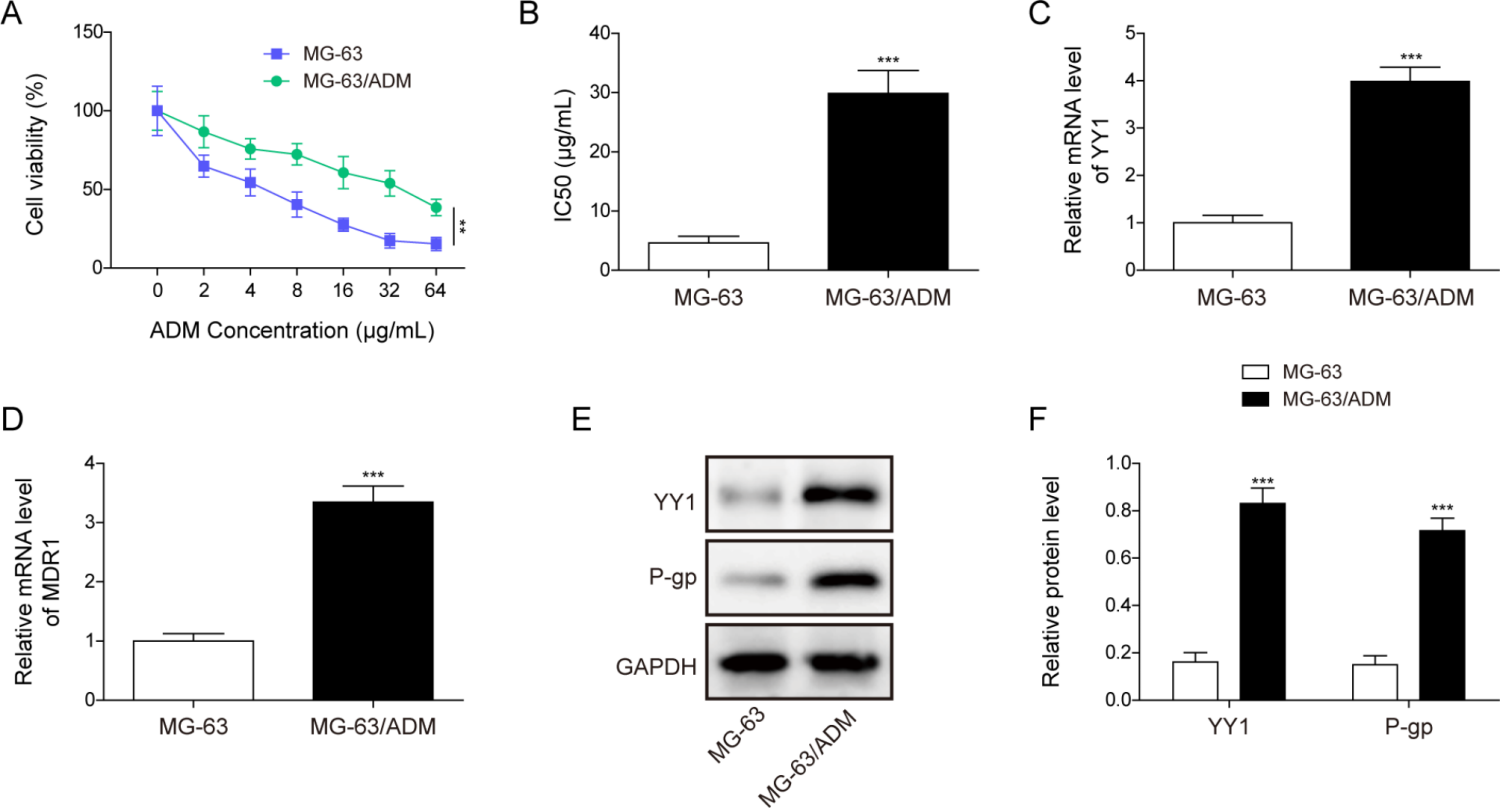
Relative expressions of YY1 and MDR1 in ADM resistant OS cells. (**A**) MG-63 and MG-63/ADM cells were treated with different concentrations of ADM ranging from 2 µg/mL to 64 µg/mL, and cell viability was assessed by MTT assay. (**B**) IC50 value was calculated in MG-63 and MG-63/ADM cells. (**C**) The mRNA expression levels of YY1 (**C**) and MDR1 (**D**) in MG-63 and MG-63/ADM cells were detected via qRT-PCR. (**E**) YY1 and P-gp protein expressions in MG-63 and MG-63/ADM cells were detected by Western blotting. (**F**) Quantitative analysis of Western blot and GAPDH as internal reference. All the results were shown as mean ± SD (n = 3), which were three separate experiments performed in triplicate. **P < 0.01, ***P < 0.001

MG-63 and MG-63/ADM cells were treated with ADM at different concentrations ranging from 2 to 64 µg/mL, and cell viability was evaluated using the MTT assay. As shown in Fig. 1A &B, ADM inhibited cell viability of OS cells in a dose-dependent manner, with IC50 value of 4.5 µg/mL and 30 µg/mL for MG-63 cells and MG-63/ADM resistant cells, respectively. The mRNA levels of YY1 and MDR1 in MG-63/ADM cells were remarkably higher than those in MG-63 cells (Fig. 1C&D). Since the P-gp protein was encoded by MDR1 gene, its expression was assessed by Western blotting. The protein levels of YY1 and P-gp in MG-63/ADM cells were markedly elevated than those in MG-63 cells (Fig. 1E&F). The above data revealed that YY1 and MDR1 expression levels were enhanced in MG-63/ADM cells.

### Met increased sensitivity of MG-63/ADM cells to ADM and accelerated cell apoptosis

**Fig. 2:**
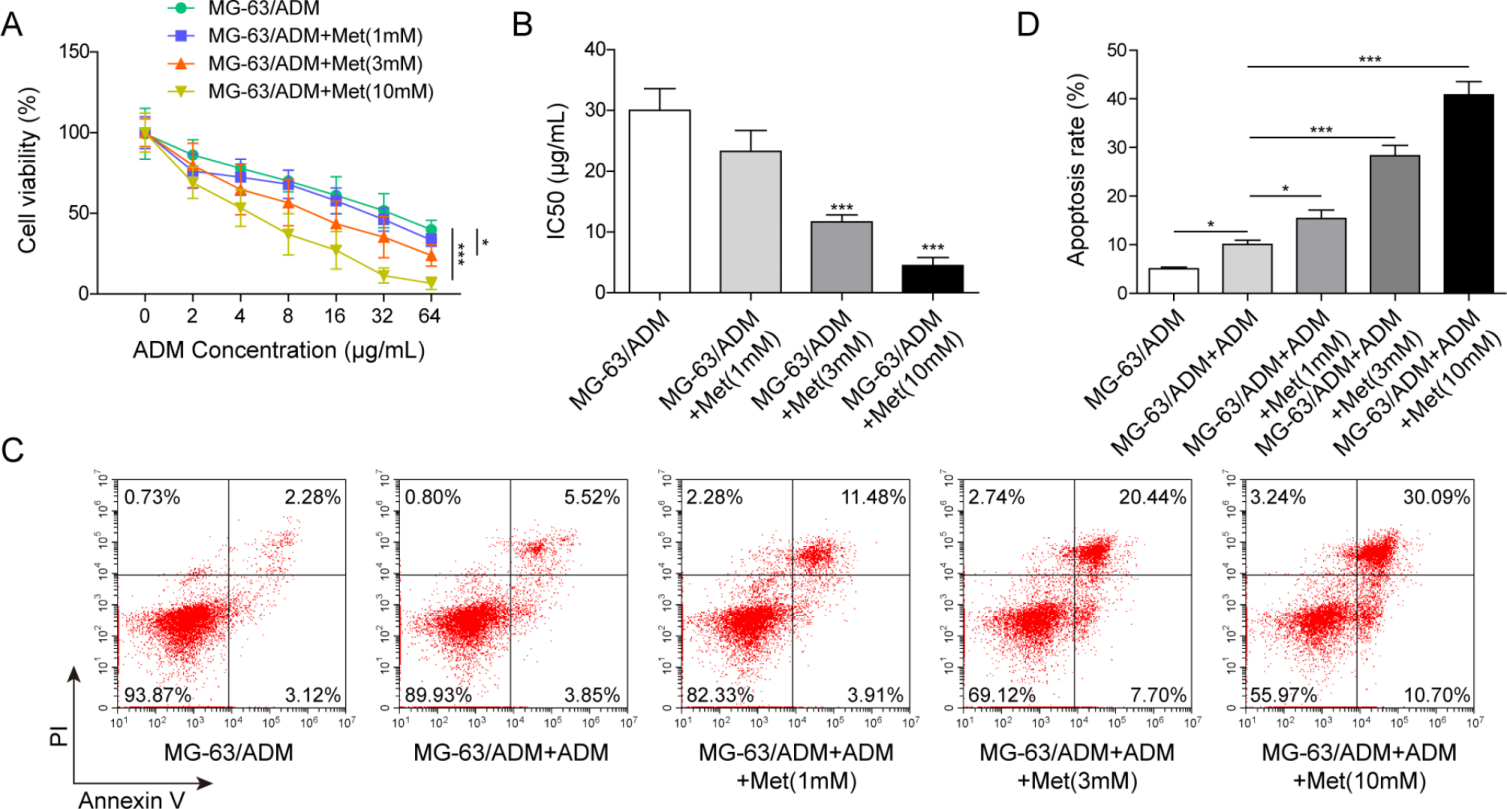
Met increased sensitivity of MG-63/ADM cells to ADM and accelerated cell apoptosis. (**A**) MG-63/ADM cells were treated with different concentrations of Met ranging from 1mM to 10 mM and cell viability was assessed by MTT assay. (**B**) IC50 value was calculated MG-63/ADM cells with Met treatment. (**C**) ADM induced apoptosis of MG-63/ADM cells after Met induction was determined by flow cytometry. (**D**) The apoptosis rate was calculated. All the results were shown as mean ± SD (n = 3), which were three separate experiments performed in triplicate. *P < 0.05, ***P < 0.001

To investigate the effect of Met on MG-63/ADM cells, MG-63/ADM cells were exposed to Met at concentrations of 1 mM, 3 mM and 10 mM. The results of MTT assay showed that Met promoted the inhibitory effects of ADM on cell viability of MG-63/ADM cells in a dose-dependent manner, with a significant decrease in IC50 values of ADM (Fig. 2A&B). Moreover, under low, medium and high concentrations of Met treatment, the effects of ADM-induced apoptosis on MG-63/ADM cell were promoted in a dose-dependent manner (Fig. 2C&D). Based on these results, Met increased the sensitivity of MG-63/ADM cells to ADM and accelerated apoptosis of MG-63/ADM cells.

### Met suppressed YY1 and MDR1 expressions in MG-63/ADM cells

**Fig. 3:**
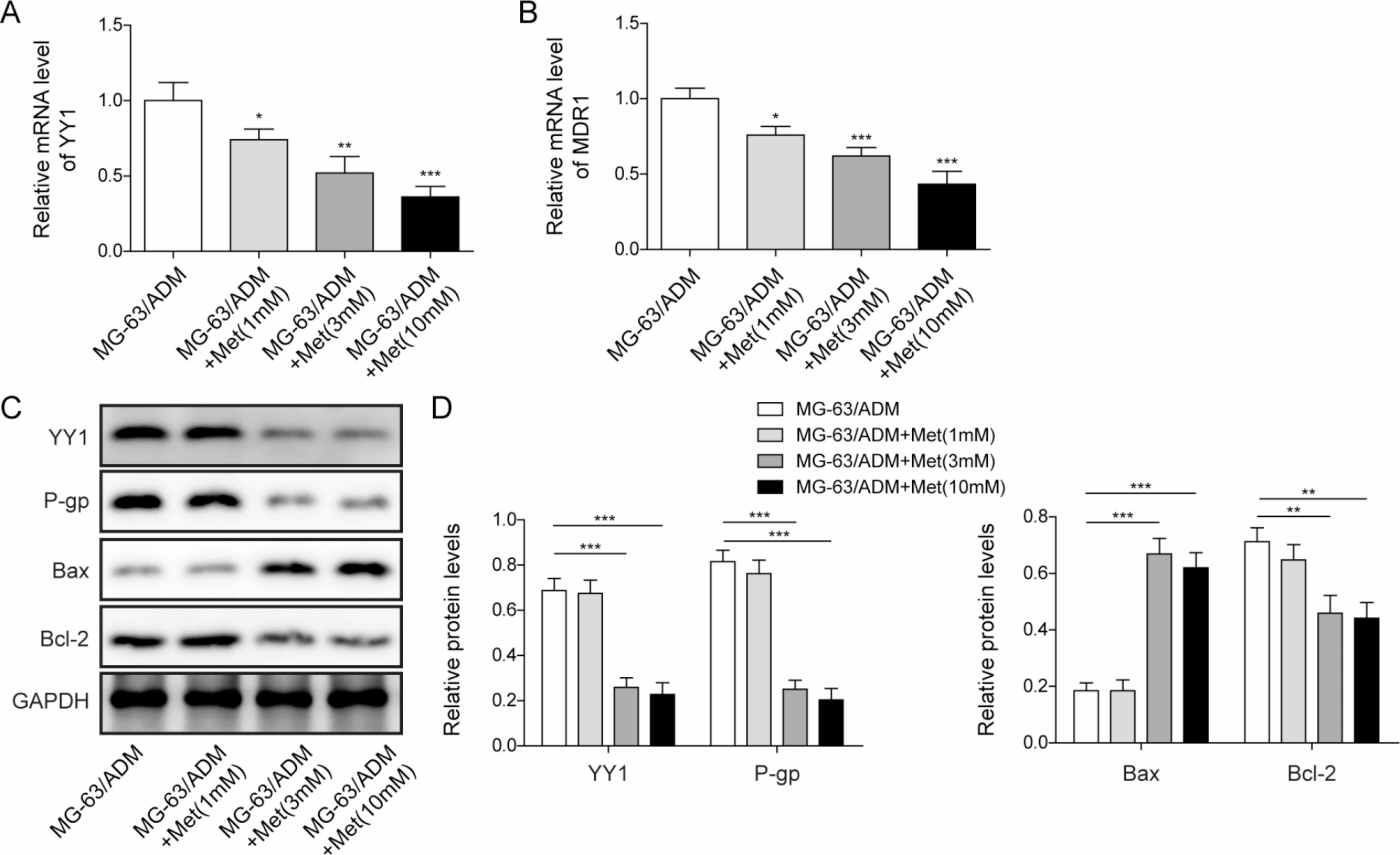
Met suppressed the expression of YY1 and MDR1 in MG-63/ADM cells. The mRNA expression levels of YY1 (**A**) and MDR1 (**B**) after Met treatment were detected via qRT-PCR. (**C**) The protein expression levels of YY1, P-gp, Bcl-2 and Bax following Met exposure were detected by Western blotting. (**D**) Quantitative analysis of Western blot and GAPDH as internal reference. All the results were shown as mean ± SD (n = 3), which were three separate experiments performed in triplicate. *P < 0.05, ***P < 0.001

To verify the effect of Met on the expression of YY1 and MDR1, MG-63/ADM cells were treated with low, medium and high concentrations of Met, respectively. Met restrained YY1 and MDR1 mRNA levels in MG-63/ADM cells in a dose-dependent manner (Fig. 3A&B). Additionally, YY1 and P-gp protein expressions in MG-63/ADM cells were suppressed by Met, and the effect was dose-dependent (Fig. 3C&D). Furthermore, Met suppressed the protein level of anti-apoptotic protein Bcl-2 and promoted the protein level of pro-apoptotic protein Bax (Fig. 3C&D). Taken together, these findings indicated that Met markedly inhibited YY1 and MDR1 levels in MG-63/ADM cells.

### YY1 promoted MDR1 expression by directly binding to its promoter

**Fig. 4:**
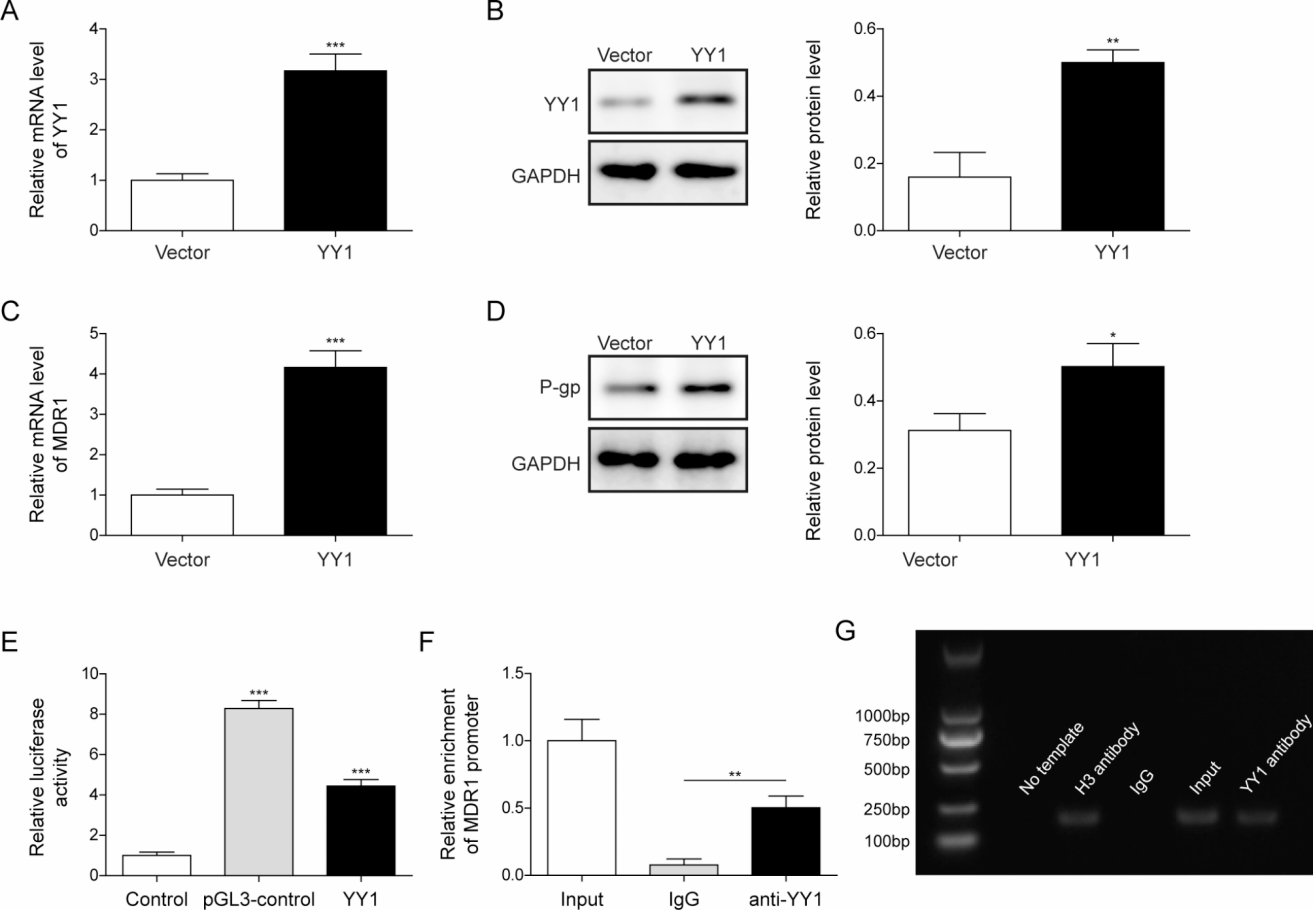
YY1 promoted MDR1 expression by directly binding to its promoter (**A**) The expression of YY1 after transfection with YY1 overexpression vector was measured via qRT-PCR. (**B**) The protein expression of YY1 after transfection with YY1 overexpression vector was detected by Western blotting. (**C**) The mRNA levels of MDR1 were measured via qRT-PCR. (**D**) P-gp protein expressions were detected by western blotting. (**E**) Dual-luciferase reporter detection was carried out to measure the transcriptional activity of MDR1 with YY1 overexpression. (**F**) The targeting relationship between YY1 and MDR1 was determined by ChIP-qPCR assay. (**G**) Agarose gel electrophoresis of ChIP-qPCR results were assayed. All the results were shown as mean ± SD (n = 3), which were three separate experiments performed in triplicate. *P < 0.05, **P < 0.01, ***P < 0.001

To further explore the mechanism of YY1 in OS resistance to ADM, MG-63/ADM cells were transfected with YY1 overexpression vector. As shown in Fig. 4A&B, transfection of YY1 overexpression vector dramatically elevated YY1 mRNA and protein expressions. Furthermore, overexpression of YY1 could markedly increase the level of MDR1 mRNA and its encoded P-gp protein (Fig. 4C&D). Dual-luciferase reporter detection was performed and showed that overexpression of YY1 could promote the transcriptional activity of MDR1 (Fig. 4E). ChIP-qPCR assay was performed to determine the targeting relationship between YY1 and MDR1 promoter. The results showed that YY1 antibody could elevate the enrichment of MDR1 promoter compared with IgG antibody (Fig. 4F). YY1 antibody enriched the MDR1 promoter region, indicating that YY1 directly bound to MDR1 promoter (Fig. 4G). The data implicated that YY1 promoted the expression of MDR1 by directly binding to its promoter.

### Overexpression of YY1 or MDR1 reversed the effect of Met on ADM sensitivity of MG-63/ADM cells

**Fig. 5:**
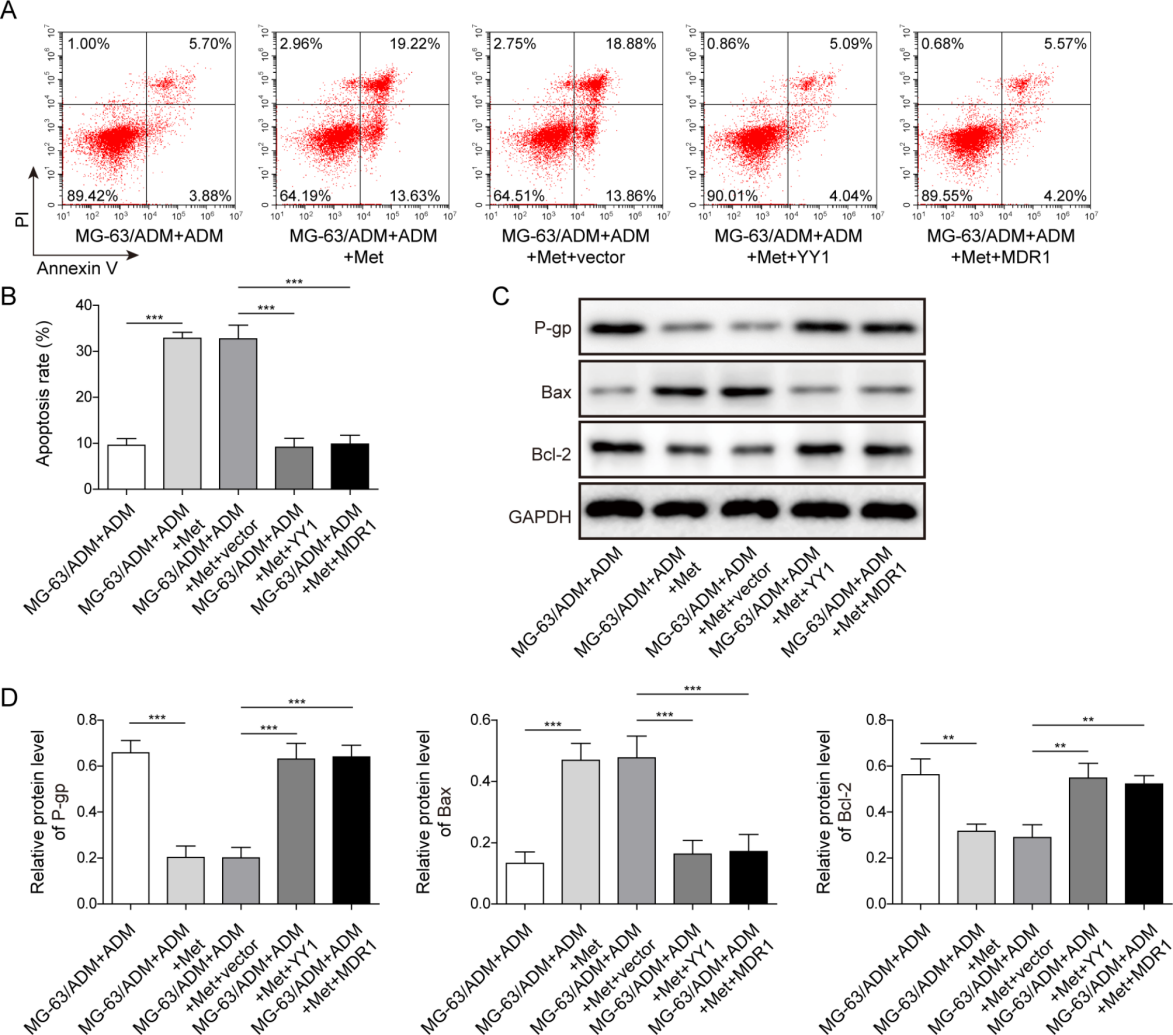
Overexpression of YY1 or MDR1 reversed the effect of Met on ADM sensitivity of MG-63/ADM cells. (**A**) The cytotoxic effects of Met treatment on ADM-induced apoptosis of MG-63/ADM cells after overexpression of YY1 or MDR1 were determined by flow cytometry. (**B**) The apoptosis rate was calculated. (**C**) Western blotting was performed to detect the protein levels P-gp, Bcl-2 and Bax in MG-63/ADM cells with YY1 or MDR1 overexpression. (**D**) Quantitative analysis of Western blot and GAPDH as internal reference. All the results were shown as mean ± SD (n = 3), which were three separate experiments performed in triplicate. **P < 0.01, ***P < 0.001

To investigate the effect of YY1 or MDR1 on Met increased sensitivity to ADM, YY1 and MDR1 overexpression vectors were constructed and transfected into MG-63/ADM cells. The results showed that the apoptosis rate of MG-63/ADM cells induced by Met combined with ADM was higher than that induced by ADM alone, while overexpression of YY1 or MDR1 could significantly reverse the apoptosis changes in Met induced apoptosis (Fig. 5A&B). In addition, Met promoted the expression of Bax and inhibited the expression of P-gp and Bcl-2 in ADM-induced MG-63/ADM cells, while overexpression of YY1 or MDR1 obviously reversed the changes induced by Met (Fig. 5C&D). Collectively, these results indicated that Met exacerbated apoptosis in MG-63/ADM cells with ADM induction, which was reversed after overexpression of YY1 or MDR1.

## Discussion

At present, the chemotherapy regimen for OS patients was mainly cisplatin, ADM, methotrexate and ifosfamide, and the combination of these anti-OS drugs improved 5-year survival rate of OS patients [21]. However, the prognosis of most OS patients with metastatic or recurrent remained poor and the odds of long-term survival remained low [22]. To some extent, this result was related to chemoresistance to anti-OS drugs. Therefore, elucidating the regulatory mechanism of chemotherapy resistance is of great significance to improve the treatment of OS. Our study indicated that Met treatment alleviated ADM resistance of OS by regulating YY1/MDR1 signaling pathway, providing a new insight into the mechanism of Met in ADM resistance of OS.

Met belongs to the biguanide family which is commonly used for treatment of type 2 diabetes mellitus [13]. Met has been proved to play an important role in anti-proliferation and apoptosis effects in a variety of cancer cells [23]. Previous studies indicated that Met could impede the development of chemoresistance via enhancing drug sensitivity, suppressing cell proliferation, and reducing migration ability of tumor cells [24]. Met eliminated chemoresistance by blocking the enhancement of Nrf2 hydroxymethylation levels [25]. It was demonstrated that Met could reverse insulin-induced oxaliplatin resistance via the AMPK/Erk pathway in human colon cancer cells [26]. Met exhibited anti-tumor activity against the ADM-resistant breast cancer MCF7/ADR cells by restraining the expression of P-gp [27]. Furthermore, Met elevated the sensitivity of OS cells to ADM and cisplatin [11], but the underlying mechanism is still unclear. It has been reported that Met increased miR-570-3p through DNA demethylation, inhibiting the expression of LCMR1 and ATG12, thereby repressing the metastasis of OS cells [28]. In this study, it was found that Met promoted the sensitivity of MG-63/ADM cells to ADM and accelerated apoptosis of MG-63/ADM cells.

P-gp is a kind of transporter encoded by MDR1 family and involves in chemoresistance of multiple tumors [23]. OS with different responses to adjuvant chemotherapy could be determined via P-gp expression at the clinical onset of tumor cells [29]. YY1 is a zinc finger structural protein belonging to the human GLIKruppel family of nuclear protein, which functions as a transcriptional activator or repressor in a variety of cancers. The overexpression of YY1 in the primary site of OS was correlated with metastasis and poor prognosis of OS patients [16]. Inhibition of YY1 could partially regulate the sensitivity of human prostate PC-3 cancer cells to TRAIL mediated apoptosis induced by chemotherapeutic drugs [30]. However, the relationship between Met and YY1 and its role in OS chemotherapy resistance remain unclear. Our findings revealed that Met suppressed the expression of YY1 and MDR1 in MG-63/ADM cells, and overexpression of YY1 could directly bind to the MDR1 promoter and significantly increase the expression level of MDR1 and its encoded P-gp protein in MG-63/ADM cells.

As reported, Met suppressed MDR1 promoter activity and protein expression by inhibiting the activation of NF-κB and CREB in breast cancer ADM resistant cells (MCF-7/ADR) [23]. Met could transcriptionally down-regulated the expression of MDR1 by modulating AMPK/mTOR pathway and inhibiting HIF-1α expression, leading to reverse multidrug resistance in hepatocellular carcinoma [31]. These reports combined with our study suggested that Met might inhibit MDR1 expression in a variety of tumor cells, rather than have a unique effect in OS. Moreover, novel curcumin analogues and (-)-Epigallocatechin gallate (EGCG) reversed MDR1-mediated multidrug resistance in paclitaxel-resistant human breast cancer cells and 5-FU-resistant colorectal cancer, respectively [32, 33], indicating that other drugs also targeted MDR1 pathway to block chemotherapy resistance. Additionally, miRNA-221 could promote ADM resistance in OS cells through activating the Stat3 pathway to increase P-gp and Bcl-2 expression [34]. Similar to the above studies, our study demonstrated that Met regulated the expression levels of apoptosis related protein Bcl-2 and Bax. Met combined with ADM accelerated the apoptosis rate and expression of pro-apoptotic proteins in MG-63/ADM cells, while overexpression of YY1 or MDR1 could significantly reverse Met induced apoptosis changes. These results suggested that YY1 or MDR1 played a key role in Met increasing the sensitivity of MG-63/ADM cells to ADM.

Although this study has achieved significant results in cell models, its feasibility and effectiveness in mouse models and clinical treatments have not yet been verified. In addition, the side effects of Met may affect its use in treatment. As an oral hypoglycemic drug, Met has common side effects including diarrhea, nausea, and vomiting. These side effects may affect the quality of life of patients and limit the use of Met in the treatment of OS. Therefore, although Met can reduce the resistance of OS to ADM, whether its resistance to other chemotherapeutic drugs for OS is effective still needs further research. Additionally, more animal and clinical studies are needed to further confirm the impact of Met on chemoresistance of OS.

## Conclusion

**Fig. 6:**
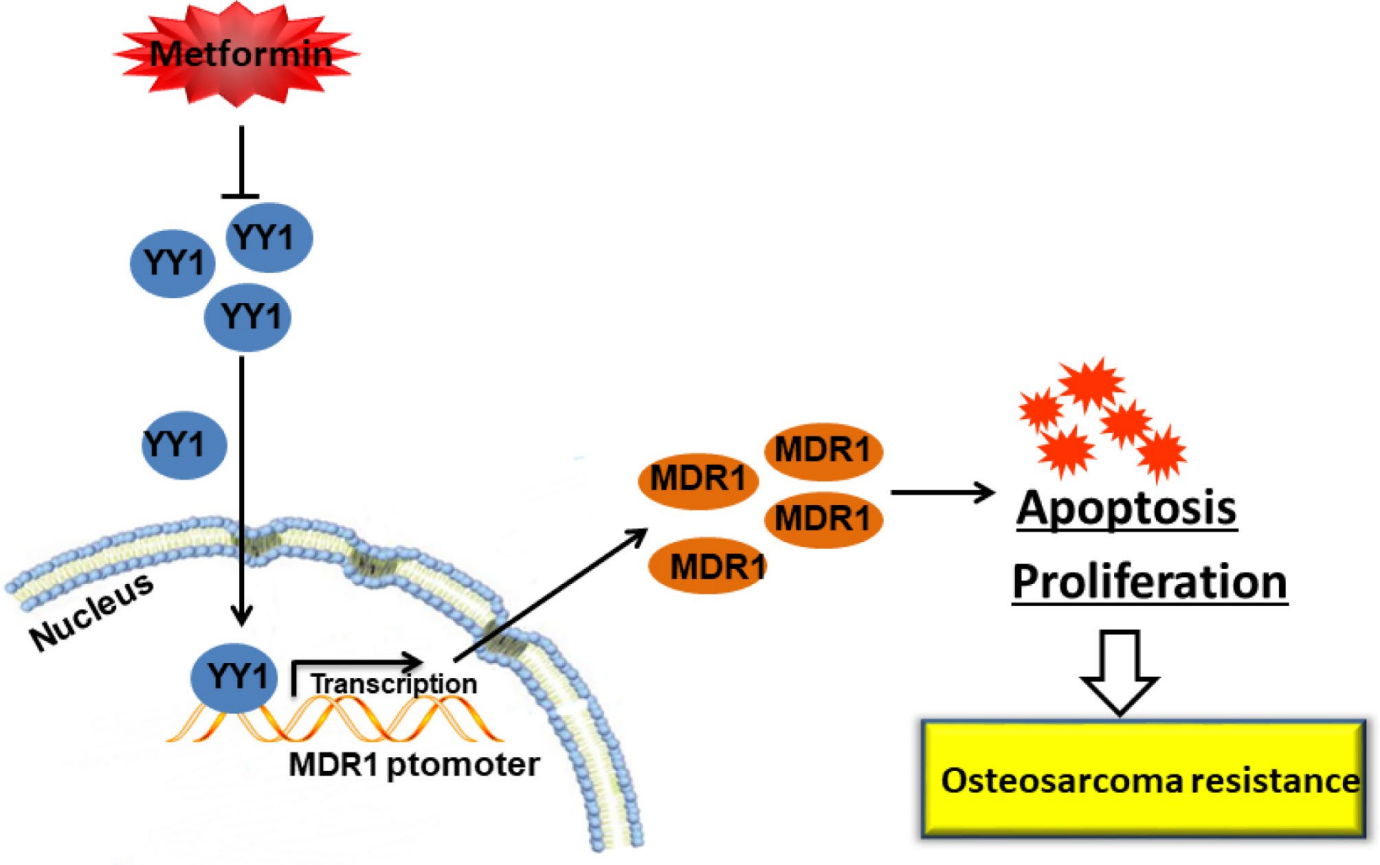
A schema of Met in OS ADM resistance. Met alleviated ADM resistance of OS by decreasing YY1 to directly inhibit transcription and expression of MDR1.

In this study, we demonstrated that Met could decrease the transcription and expression of multidrug resistance gene MDR1 by down-regulating transcription factor YY1, thereby inhibiting the resistance of OS to ADM (Fig. 6). Met, as a potential drug for the treatment of OS, has a broad research prospect. Although this study is still in its preliminary stage, it provides new ideas for the treatment of OS drug resistance and provides the possibility of developing new treatment options.

### Electronic supplementary material

Below is the link to the electronic supplementary material.

Supplementary Material 1

## Data Availability

Data sharing not applicable to this article as no datasets were generated or analysed during the current study.
