# Supplementary figures and images for: Metformin alleviates adriamycin resistance of osteosarcoma by declining YY1 to inhibit MDR1 transcriptional activity
Source: BMC Pharmacol Toxicol. 2023 Oct 12;24:50. doi: 10.1186/s40360-023-00685-8 (PMC10571298; doi:10.1186/s40360-023-00685-8)

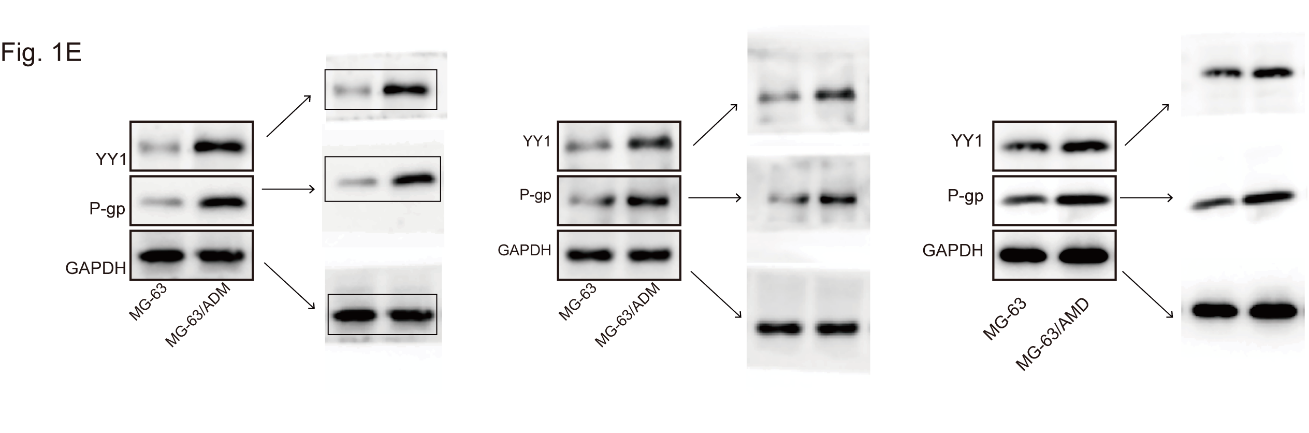


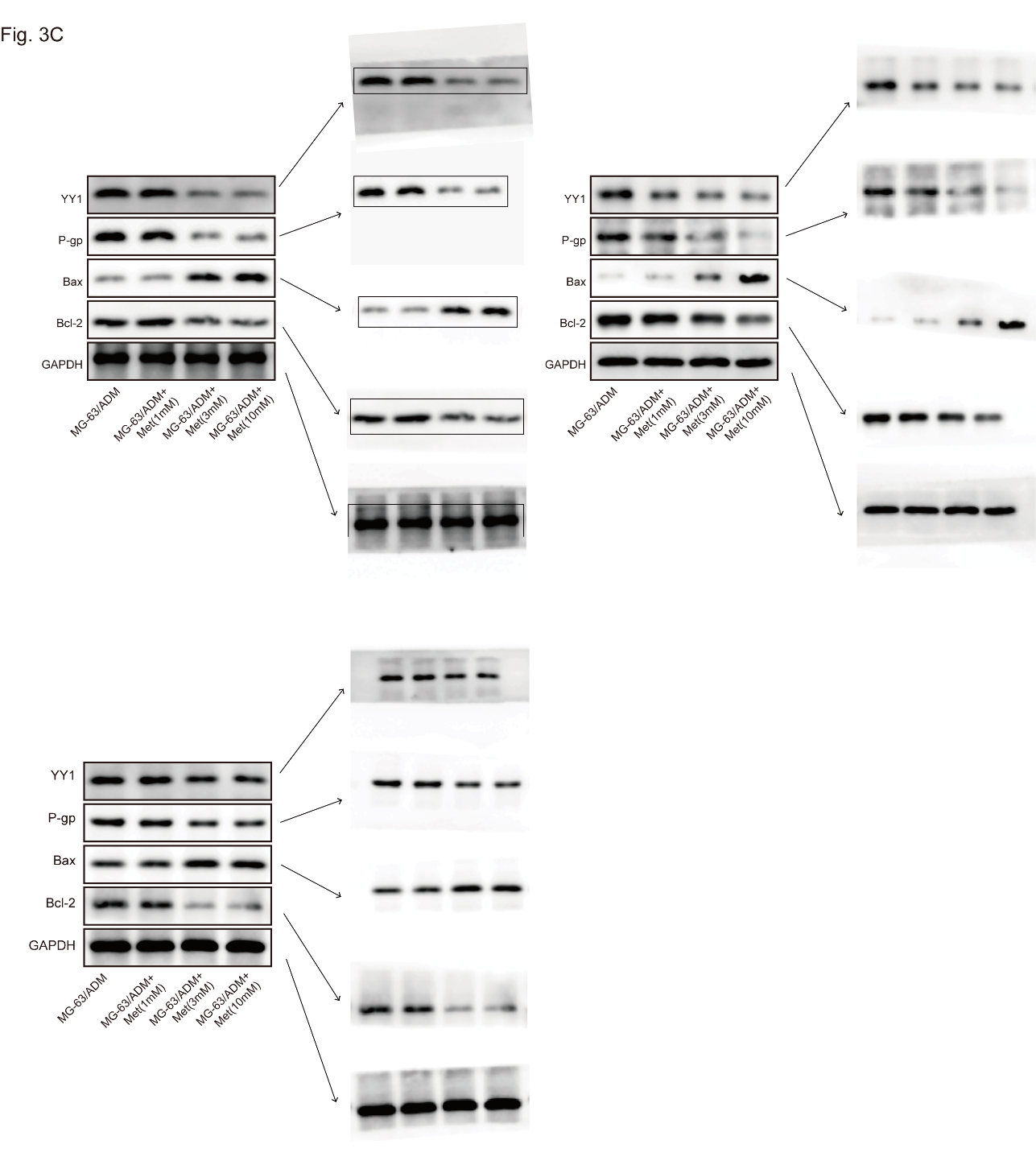


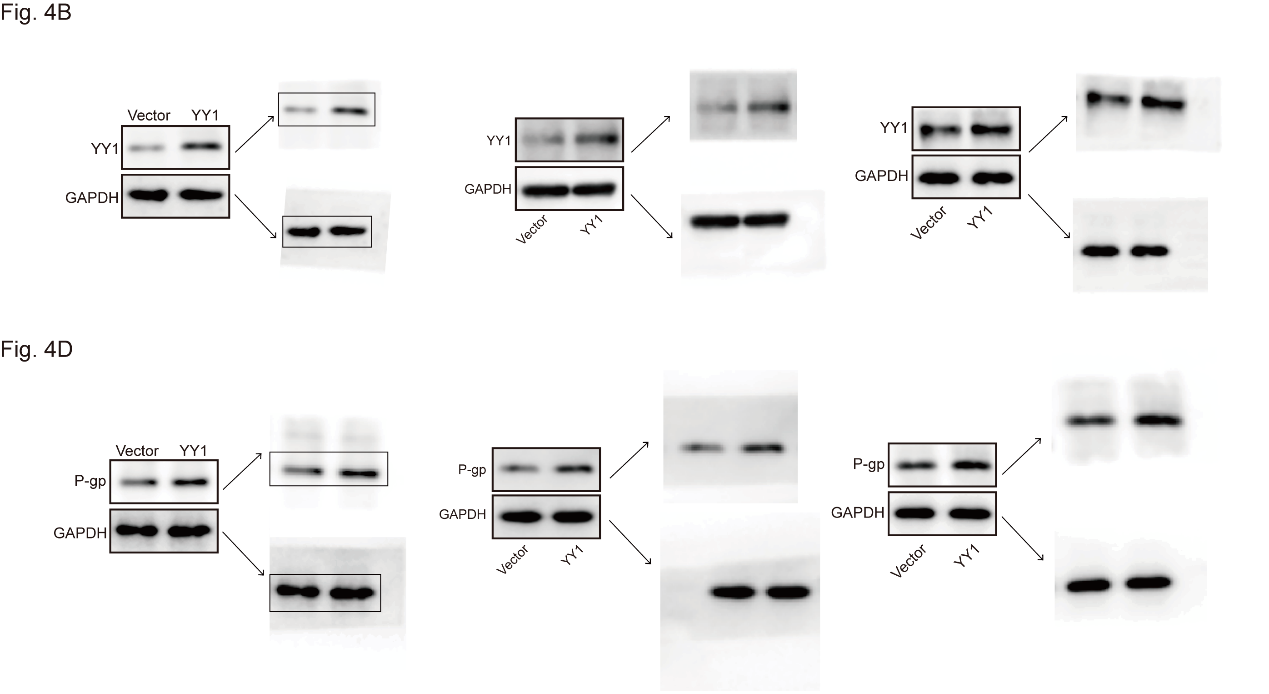


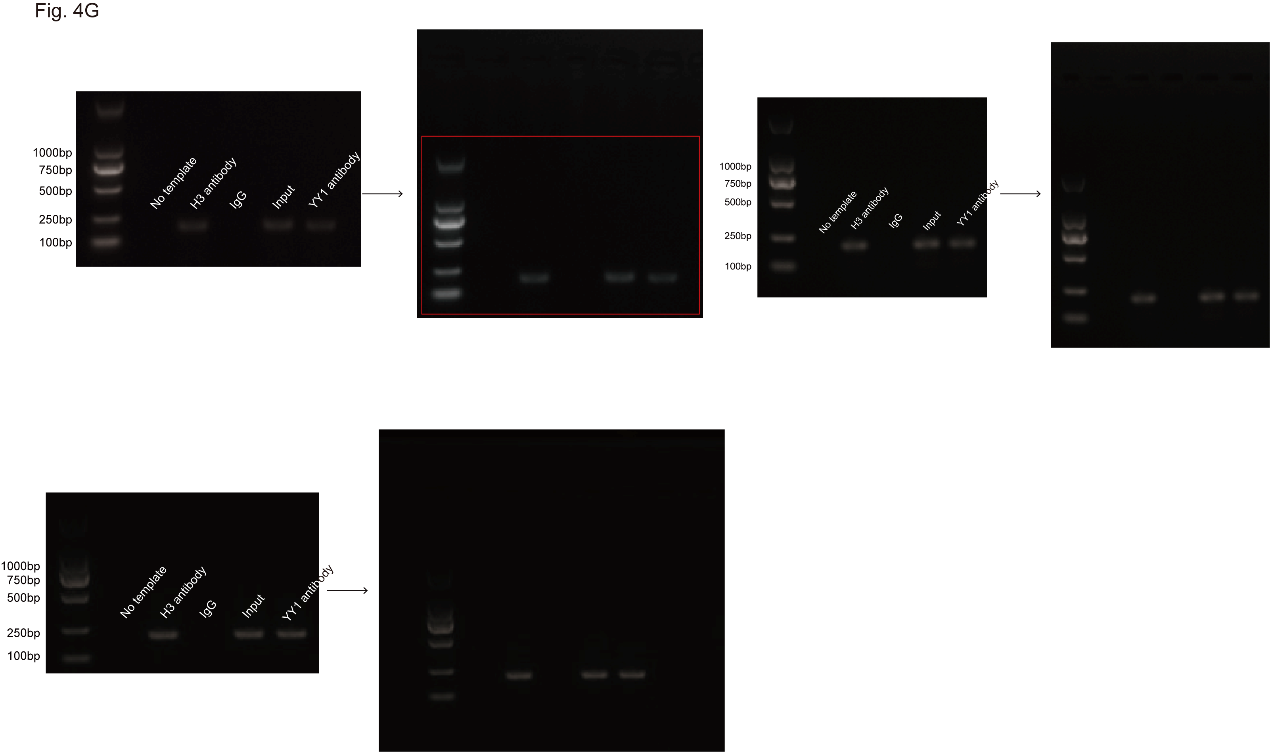


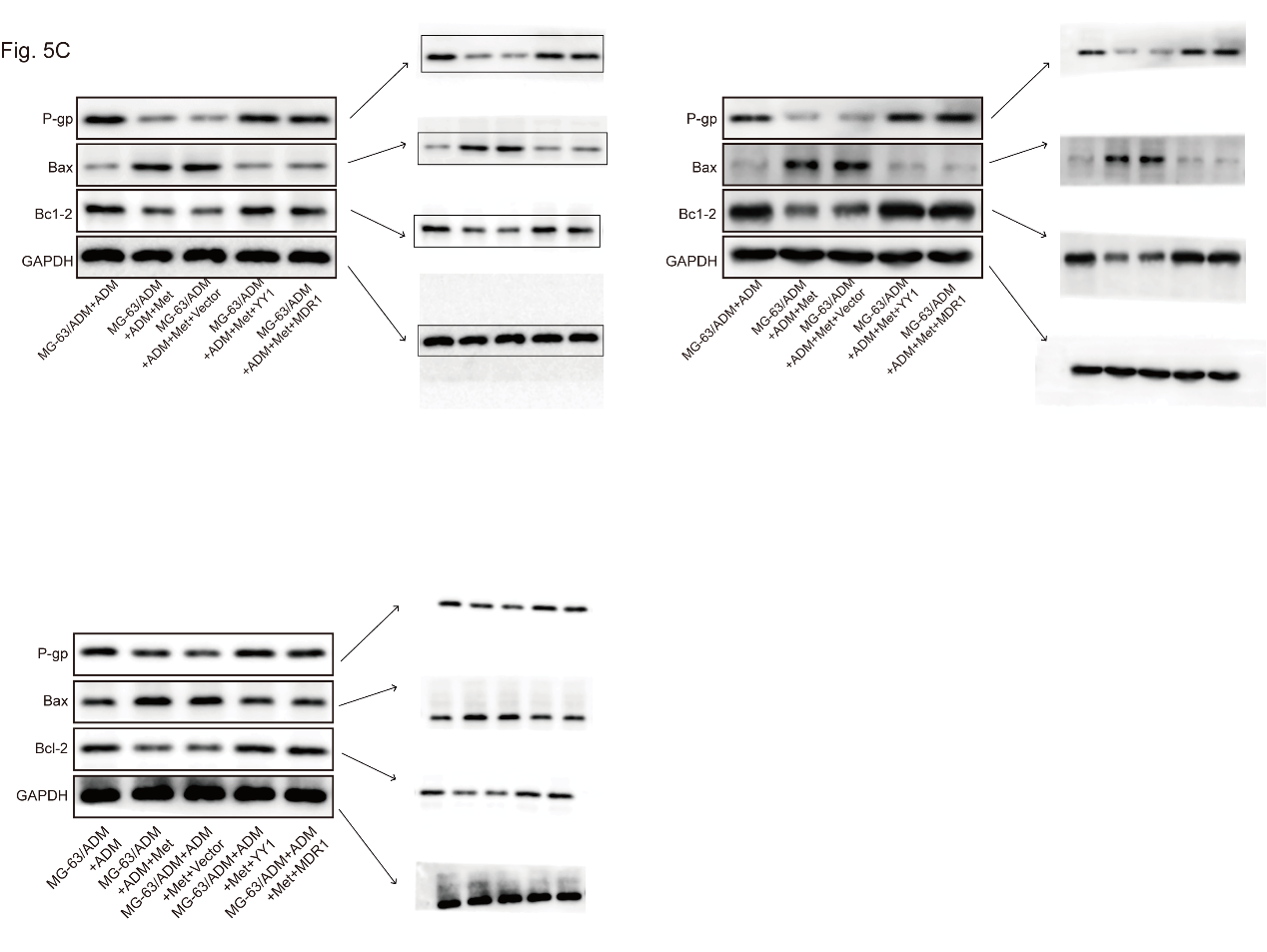

Supplement: Supplementary file 1 — Supplementary Material 1 [file 40360_2023_685_MOESM1_ESM.docx]
